# Vitamin D Vitamers Affect Vitamin D Status Differently in Young Healthy Males

**DOI:** 10.3390/nu10010012

**Published:** 2017-12-23

**Authors:** Jette Jakobsen, Elisabeth Anne Wreford Andersen, Tue Christensen, Rikke Andersen, Susanne Bügel

**Affiliations:** 1 National Food Institute, Technical University of Denmark, 2800 Lyngby, Denmark; tuchr@food.dtu.dk (T.C.); rian@food.dtu.dk (R.A.); 2 Institute of Mathematics and Computer Science, Technical University of Denmark, 2800 Lyngby, Denmark; elian@cancer.dk; 3 Department of Nutrition, Exercise and Sports, University of Copenhagen, 1958 Frederiksberg C, Denmark; shb@nexs.ku.dk

**Keywords:** 25-hydroxyvitamin D3, vitamin D2, vitamin D3, humans, bioactivity, supplements

## Abstract

Dietary intake of vitamin D includes vitamin D3 (vitD3), 25-hydroxyvitamin D3 (25OH-D3), and vitamin D2 (vitD2). However, the bioactivity of the different species has not been scientifically established. The hypothesis in this study was that vitD3, 25OH-D3, and vitD2 have an equal effect on 25-hydroxyvitamin D in serum (vitamin D status). To test our hypothesis, we performed a randomized, crossover study. Twelve young males consumed 10 µg/day vitD3 during a four-week run-in period, followed by 3 × 6 weeks of 10 µg/day vitD3, 10 µg/day 25OH-D3, and 10 µg/day vitD2. The content of vitD3, vitD2, 25OH-D3, and 25-hydroxyvitamin D2 (25OH-D2) in serum was quantified by liquid chromatography-tandem mass spectrometry (LC-MS/MS). The hypothesis that the three sources of vitamin D affect vitamin D status equally was rejected. Based on the assumption that 1 µg vitD3/day will show an increase in vitamin D status of 1.96 nmol/L, the results showed that 23 µg vitD2 and 6.8 µg 25OH-D3 was similar to 10 µg vitD3. These results demonstrate that further investigations are necessary to determine how to quantify the total vitamin D activity based on chemical quantification of the individual vitamin D metabolites to replace the total vitamin D activity assessed in biological rat models.

## 1. Introduction

Dietary intake of vitamin D includes the parent forms vitamin D3 (vitD3) and vitamin D2 (vitD2), and the hydroxylated forms 25-hydroxyvitamin D3 (25OH-D3) and 25-hydroxyvitamin D2 (25OH-D2). VitD3 and 25OH-D3 are found in fish, eggs, meat, and dairy products [1], vitD2 is found in wild mushrooms, whereas beef and dairy products contain vitD2 and 25OH-D2 [2,3]. To calculate the total vitamin D activity in food conversion factors between the different vitamin D forms are essential. However, the contribution of the different forms to the total vitamin D activity is a topic of controversy [4,5,6,7,8,9].

Studies that have compared the effect of dietary intake of vitD3 and vitD2 on the vitamin D status have been evaluated in a systematic review and meta-analysis [6]. The overall conclusion was that when vitamin D was administered once or as a monthly bolus, vitD3 was superior to vitD2 in increasing the vitamin D status [10,11,12], whereas no difference in the vitamin D status was observed if vitD2 and vitD3 were administered on a daily basis [10,13,14,15,16]. Investigation of 25OH-D2 half-life versus 25OH-D3 half-life showed no difference in participants from the UK, i.e., 15.1 and 15.6 days, respectively, while a difference was found in participants from Gambia, i.e., 12.8 and 14.7 days. Furthermore, half-lives were affected by the concentration and genotype of vitamin D binding protein [17]. There have been fewer studies that have investigated the difference in bioactivity between the oral intake of vitD3 and 25OH-D3. In a review, it was concluded that, in rat models, the conversion factor for the content of 25OH-D3 to vitD3 is between one and five. Based on calcification score testing in rachitic rats, the biological activity of 25OHD was between one and two times greater than that of vitamin D, whereas a factor of five was determined by using intestinal absorption of calcium, which is not an accepted clinical endpoint parameter [7]. Feeding studies in slaughter pigs, which were fed vitD3 and 25OH-D3 daily from weaning until they were slaughtered, showed that the efficacy of the two metabolites to increase the vitamin D status was equal [18], whereas other studies in pigs with daily supplementation have shown 25OH-D3 to be 2–3 times more efficient than VitD3 in increasing the vitamin D status [19,20,21]. However, the conversion factors for vitamin D metabolites compared to vitD3 should preferably be based on studies in humans. Until now, only a few randomized controlled studies with daily supplementation have been conducted in humans, where different study design and calculation strategies to establish the conversion factor to be between two and five have been applied [8,9,22].

Jakobsen et al. (2009) reported the results from four individual randomized controlled trials (RCTs) in post-menopausal women using a run-in period of four to eight weeks followed by daily supplementation of 5–10 µg of vitD3 for 16 weeks to 20 months [23]. Within the individual subjects, no significant difference in vitamin D status was observed after the run-in period until the end of the intervention, but individual vitamin D status showed a large variation and ranged from 48 nmol/L to 120 nmol/L.

The increase in vitamin D status by daily supplementation has been shown to be curvilinear [24,25]. Individual studies estimated the increase to be 0.70 nmol/L for each 1 µg of dietary intake of vitD3 based on supplementation of 0–250 µg vitD3/day in a study conducted in Omaha, NE, USA at 41.2° N latitude [26], but 1.96 nmol/L for each 1 µg of vitD3 based on supplementation of 0–15 µg vitD3/day in a study conducted in Ireland at 51–54° N latitude [27]. Based on a careful selection of studies in which 5 to 50 µg of vitD3 was administered daily, it was concluded that 1 µg vitD3 increases the vitamin D status by 2 nmol/L [28].

The aim of this human intervention study was to investigate if equal amounts of vitD3, vitD2, and 25OH-D3 given as supplements exhibit equal bioactivity, measured as 25-hydroxyvitamin D in serum (S-25OHD), in healthy males aged 20–30 years in a randomized crossover design. Furthermore, if the hypothesis was not accepted, the aim was to assess the differences in bioactivity between vitD3, vitD2, and 25OH-D3.

## 2. Materials and Methods

### 2.1. Randomized Controlled Trial

A total of 12 healthy, free-living male adults aged 20–30 years were recruited in this 3 × 6 weeks vitamin D intervention trial. The subjects were recruited among students from the University of Copenhagen through the use of advertisements placed around the university campus. Volunteers were excluded if they had a BMI > 27 kg/m^2^, had donated blood within the last three months, had any chronic diseases, used medication regularly except for the occasional use of painkillers, were hypercalcemic, consumed an excessive amount of alcohol, or had known malabsorption syndromes. Furthermore, to decrease sun exposure, volunteers who planned to go skiing or travel south of 55° N during the duration of the study were excluded. All subjects were Caucasian, had low habitual fish intake (less than twice a week), and were non-smokers. At screening all subjects were instructed to maintain the same level of physical activity throughout the study and agreed to refrain from donating blood, as well as from taking any kind of vitamin, mineral, or dietary supplement other than supplements provided in the study. All subjects also agreed to abstain from going to a solarium during the intervention.

The study was approved by the Local Research Ethics Committee of Copenhagen and Frederiksberg. All participants gave their written informed consent according to the Helsinki Declaration. This trial was registered as number KH 01 322182 (www.clinicaltrials.gov; KH 01 322182).

#### 2.1.1. Rationale and Design of Study

The present study was designed as a double-blind randomized crossover trial in which adult males were assigned to receive tablets containing 10 µg vitD3, 10 µg vitD2, and 10 µg 25OH-D3 daily in a random order. Prior to the intervention, all subjects received 10 µg vitD3 daily for four weeks to achieve a steady state in vitamin D status.

#### 2.1.2. Tablets for RCT

The vitamin D tablets were produced at Viminco A/S, Skælskør, Denmark from the standards 1.25% vitamin D3 (Rowimix, 0440140638, lot UEC0605026, DSM Nutritional Products, Brøndby, Denmark), 100% vitamin D2 (10233619, batch 095K1306, Sigma-Aldrich, Steinheim, Germany), and 1.25% HY-D (Rowimix, 5002370360, lot WB06605237, DSM Nutritional Products, Brøndby, Denmark). Vitamin D was initially diluted into ethanol. Using cellulose and magnesium stearate as biocidal products, tablets with a diameter of 10 mm and a weight of 300 mg (283–307 mg) were formed. Each tablet contained 10 µg of vitD3, vitD2, or 25OH-D3. The tablets were stored at a maximum temperature of 5 °C until they were distributed to the subjects.

#### 2.1.3. Conduct of the Study

The study was carried out in Copenhagen, Denmark (latitude: 55° N). All subjects were recruited in September 2006, the run-in period started in mid-October, and the study was finished at the end of March 2007. During the study, the blood samples from subjects were collected five times; before run-in, at baseline (day 1), and at end of each of the six weeks period (day 71, day 113, and day 155). Blood samples were drawn by a trained medical laboratory technician in the morning (between 8:00 a.m. and 9:00 a.m.) after 12 h of fasting with allowance to drink up to 1/2 L of water. The subjects were informed not to drink any kind of alcohol and to abstain from hard physical work 24 h before each blood sampling. Blood was collected by venipuncture into 10 mL dry tubes (BD vacutainer, ref. no. 368430, Becton Dickenson, Franklin Lakes, NJ, USA) for analysis of the serum vitamin D level, in 5 mL tubes (vacutainer, ref. no. 367614, Becton Dickenson, Franklin Lakes, NJ, USA) for analysis of the serum parathyroid hormone (PTH) level and in 7 mL trace-element free tubes (Vacutainer, ref. no. 368380, Becton Dickenson, Franklin Lakes, NJ, USA) for analysis of calcium. Blood samples were kept at 20 °C and centrifuged after 40 min at 3000× *g* for 15 min. The serum was then transferred into plastic tubes and stored at −80 °C for vitamin D analysis or at −20 °C for serum calcium and serum PTH analysis. Anthropometric measurements (height and weight), were taken at day 1 and day 155. Body weight was measured to the nearest 0.1 kg with an electronic digital scale (Lindeltronic 8000, Lindells, Sweden). The subjects were only wearing underwear and were asked to empty their bladder before their weight was measured. The height of the subjects was measured to the nearest cm with the subject standing without shoes, gathering their feet, and head straight out in horizontal plane. Habitual intake of vitamin D and calcium was estimated by using a standardized food-frequency questionnaire that ascertained the food (incl. fortified foods) contributing to 95% of the vitamin D intake and 75% of the calcium intake [29] at screening, day 57, and day 155. A health and lifestyle questionnaire, which assessed habitual fish intake, physical activity, general health, smoking status, and alcohol consumption, was completed at screening. Compliance was assessed by counting the tablets.

### 2.2. Laboratory Analysis

#### 2.2.1. Vitamin D in Tablets

The content of vitamin D compounds in the tablets was analyzed four times during the intervention, at screening and after two, four, and five months. Briefly, five tablets were ground in a mortar and 1 g was saponified, followed by clean-up using silica solid-phase extraction and cyano-silica preparative high-performance liquid chromatography. The separated compounds were detected by reverse-phase high-performance liquid chromatography (HPLC) coupled to a diode array detector (DAD) and quantified by an internal standard method [18]. The analyses were run in a laboratory accredited according to ISO17025.

#### 2.2.2. Serum Vitamin D Metabolites

A previously described liquid chromatography-tandem mass spectrometry (LC-MS/MS) method for serum vitD3 (S-vitD3) and serum 25OH-D3 (S-25OHD3) [30] was modified to include quantification of serum vitD2 (S-vitD2) and serum 25OH-D2 (S-25OHD2). The standards used for vitD3, 25OH-D3, vitD2, and 25OH-D2 were from Sigma–Aldrich (Steinheim, Germany), whereas the deuterated internal standards 26,26,26,27,27,27-*d*_6_-vitamin D3 (*d*_6_-vitD3), 26,26,26,27,27,27-*d*_6_-25OH-D3 (*d*_6_-25OH-D3), and 26,26,26,27,27,27-*d*_6_-vitamin D2 (*d*_6_-vitD2) were from Chemaphor Inc. (Ottawa, ON, Canada), and 25,26,27-^13^*C*_3_-25OH-D2 (C_3_-25OH-D2) from IsoSciences (King of Prussia, PA, USA). In short, the protein was precipitated by the addition of acetonitrile followed by centrifugation and solid phase extraction on a HybridSPE (Supelco Analytical, Bellefonte, PA, USA). The vitamers were derivatized with 4-phenyl-1,2,4-triazoline-3,5-dione (Sigma–Aldrich, Steinheim, Germany) to enhance sensitivity and selectivity on the mass spectrometer. The precursor and product ion was *m*/*z* 591.4 → 298.0 for vitD3, *m*/*z* 597.4 → 298.0 for *d*_6_-vitD3, *m*/*z* 607.4 → 298.0 for 25OH-D3, *m*/*z* 613.4 → 298.0 for *d*_6_-25OH-D3, *m*/*z* 603.4 → 298.0 for vitD2, and *m*/*z* 609.4 → 298.0 for *d*_6_-vitD2, and *m*/*z* 619.4 → 298.0 for 25OH-D2, *m*/*z* 622.4 → 298.0 for *C*_3_-25OH-D2. Accuracy was assured by using a certified reference material (human plasma 1950, NIST, Gaithersburg, MD, USA), whereas a house-reference serum was used to control for consistency during the study. The precision (CV%) for S-vitD3, S-25OH-D3, S-vitD2, and S-25OHD-2 were 5.0%, 5.0%, 3.7%, and 2.5%, respectively.

The analyses for vitamin D metabolites were run in 2007 by a HPLC-DAD method, but repeated in October 2014 by the more specific and precise LC- MS/MS-method. Only the results from the LC-MS/MS method are presented.

#### 2.2.3. Serum Intact Parathyroid Hormone

Serum PTH was measured within three months from sampling using a solid-phase, two-site chemiluminescent enzyme-labelled immunometric assay (IMMULITE 2500 intact PTH, Diagnostic Products Corporation, Los Angeles, CA, USA). Intra- and inter-assay precision of the analysis were CV% = 5.7 and CV% = 6.3, respectively, and the reference range was 1.12–7.06 pmol/L.

#### 2.2.4. Serum Total Calcium

Serum total calcium was measured photometrically (Pentra 400, Horiba ABZ, Montpellier, France). Seronorm™ Trace Element Serum L-1 (ref 201405, SERO AS, Billingstad, Norway) was used as an external standard. The measured value 2.53 ± 1.13 mmol/L was within the specified range: 2.37–2.67 mmol/L

### 2.3. Statistical Analysis

This was a three-period, three-treatment crossover study of vitamin D in serum in which 12 healthy men received the three treatments at three time periods. Descriptive statistics were calculated for baseline results and by treatment. The results were presented as mean and standard deviation.

For the main analyses, linear models with a random person effect were used. The random person effect was included to take into account that each person had three post baseline measurements taken. Apart from the random person effect, the models included the fixed factors: treatment (vitD3, vitD2, or 25OH-D3) and period (three periods) and also the baseline level of the outcome of interest. The assumptions underlying the models (variance homogeneity and normal residuals) were checked using residual plots and normal QQ-plots and showed that the outcomes should be transformed using logarithms. However, the results were back-transformed and presented on the original scale as an estimated level with 95% confidence interval. For each outcome, an overall treatment effect was tested based on the linear mixed model followed by pairwise comparisons of the three treatments using Sidak’s method to adjust for multiple comparisons. As this was a crossover study, a possible carry-over effect was tested by including the interaction between the period and treatment in the models, but none of these interactions were statistically significant (*p*-value: 0.3–0.8) so, based on these data, there was no evidence of a carry-over effect.

The uncertainty budgets for the relative effectiveness for vitD2 compared to vitD3 and for 25OHD3 compared to vitD3 are shown in Supplementary Materials Table S2 and Table S3, respectively.

All statistical analyses were carried out using Stata v. 13 (StataCorp LP, College Station, TX, USA).

## 3. Results

### 3.1. Characteristics of the Subjects

The anthropometric data of the twelve men included in the intervention and their dietary intake of vitamin D and calcium are presented in Table 1. The body weight of the subjects did not change significantly during the study. The highest weight change was an increase of 4 kg, which was explained by a decrease in physical activity, but no change in eating habits. The subjects did not change their diet throughout the intervention and kept fish intake at a maximum of twice a week and abstained from taking vitamin supplements and going to a solarium.

The amount of vitamin D in the tablets was tested for stability (*n* = 4). No changes were identified for the three types of tablets, and the results 9.9 µg vitD3/tablet, 10.2 µg vitD2/tablet, and 9.8 µg 25OH-D3/tablet, showed no deviation from the nominal content of 10 µg/tablet.

The twelve men were carefully selected and exhibited strict complianc with experimental protocol, resulting in no missed sampling during the 22-weeks intervention study. The compliance throughout the study was 97%.

The mean dietary intake of vitamin D and calcium are listed in Table 1. A significant difference was observed between the dietary intake of vitamin D and calcium among the subjects (*p* < 0.001).

The vitamin D status before and after the run-in period showed individual effect for the 12 subjects from an increase by 23% to a decrease by 20%. Data shown in Supplementary Materials Table S1.

### 3.2. Effects of Intervention with Different Vitamin D Vitamers

In Table 2, the measured content in serum of vitamin D metabolites, PTH, and calcium is listed. The “Total 25OH-D” is the sum of S-25OH-D3 and S-25OH-D2, i.e., vitamin D status. Furthermore, the estimated levels of the same compounds are listed in Table 3. In the Supplementary Materials all individually-measured data for S-25OH-D is graphically shown in Figures S1–S3.

The results in Table 3 indicate that the level of S-25OHD3 was significantly different (*p* < 0.001) after six weeks of treatment with vitD3, vitD2, or 25OH-D3. Treatment with vitD2 resulted in the lowest level of S-25OHD3, whereas treatment with 25OH-D3 gave the highest vitamin D status. The three treatments also led to significantly different levels of S-25OHD2 (*p* < 0.001). Here, the treatment with vitD2 gave the highest level of S-25OHD2, whereas the effect of vitD3 and 25OH-D3 were very similar. The effect of the treatment on the total amount of S-25OHD was very similar to the effect on S-25OHD3.

The three treatments also affected S-vitD3 and S-vitD2. The treatment with vitD3 led to a significantly higher level of S-vitD3 compared with those from the two other treatments. The treatment with vitD2 led to the highest level of S-vitD2. The effect of the treatment was not statistically significant for serum calcium (*p* = 0.96). For serum PTH, a significant difference (*p* = 0.035) was observed, but all results were within the reference range (1.12–7.06).

### 3.3. Relative Effectiveness of Vitamin D Vitamers to Increase Vitamin D Status

The difference in vitamin status at the end of each of the intervention periods was converted into an equivalent amount of vitD3. In Table 3, the estimated levels of vitamin D status are given as 54.4 nmol/L, 43.5 nmol/L, and 63.8 nmol/L for vitD3, vitD2, or 25OH-D3, respectively. Based on an estimated increase of 1.96 nmol/L of 1 µg vitD3 [27], the decrease from 54.4 nmol/L to 43.5 nmol/L by daily supplementation of 10 µg vitD2 is similar to a dietary intake of 4.44 ± 0.56 µg vitD3, and the increase from 54.4 nmol/L to 63.8 nmol/L by daily supplementation of 10 µg 25OH-D3 can be estimated as similar to a dietary intake of 14.8 ± 1.9 µg vitD3. Compared to vitD3, the conversion factor of vitD2 and 25OH-D3 was estimated to be 0.44 and 1.5, respectively, which is equivalent to the estimation that dietary intake of 10 µg VitD3, 6.8 µg 25OH-D3, or 23 µg VitD2 will result in a similar increase of the vitamin D status.

## 4. Discussion

Due to limited data on the relative effectiveness of dietary vitD2 and 25OH-D3 compared to vitD3, we compared the effects of daily supplementation with vitD3, vitD2, and 25OH-D3 in maintaining serum 25OHD after an initial run-in period of four weeks with vitD3 to establish a steady state, in a 3 × 6 weeks double-blind, randomized, crossover trial in 12 healthy Caucasian males aged 20–30 years. We observed a significant difference between supplementation with vitD3, vitD2, or 25OH-D3 at a daily intake of 10 µg over six weeks.

An estimated increase of 0.70 nmol/L per 1 µg vitD3 was based on a daily supplementation between 25 µg to 250 µg vitD3 [26], whereas an estimated increase of 1.96 nmol/L per 1 µg vitD3 was obtained based on a daily supplementation between 5 µg to 15 µg of vitD3 [27]. A curvilinear dose response for vitamin D status has been shown in postmenopausal women supplemented daily with 10 µg to 120 µg of vitD3 [25]. Furthermore, a comprehensive study evaluating 41 studies that investigated daily supplementation of 5 µg to 50 µg found that for every extra 1 µg vitD3/day vitamin D status increased by 2.1 nmol/L (95%CI: 1.8–2.5 nmol/L) [24]. In our study, we used a similar daily supplementation level as Cashman et al. (2008), which was the reason we used 1.96 nmol/L in our estimation [27].

A comparison to other studies investigating the potential difference between vitD3, vitD2, and 25OH-D3 should be done with caution owing to large differences in study designs. Except for Logan et al. (2013), who aimed to maintain a steady vitamin D status [31], all other study designs have focused on the ability of the different vitamin D species to increase S-25OHD. Daily supplementation with 5–25 µg vitD3 does not necessarily increase vitamin D status to the same level, e.g., 15 µg vitD3/day showed both an increase [32,33] and a decrease [27] in vitamin D status. Potential sources of error are the difference in the analytical methods applied in the different studies [23,34,35] and the difference in the setup of the intervention studies, e.g., vitamin D status at the start, which influences the increase in the vitamin D status [36].

A meta-analysis identified no difference in the vitamin D status when vitD3 and vitD2 supplementation was given on a daily basis [6]. Not included in the meta-analysis is a long-term study over a period of 25 weeks with daily supplementation of 25 µg of vitD2 and vitD3, which resulted in lower vitamin D status in the vitD2 group than in the vitD3 group [31]. Furthermore, an alternative estimation method is to calculate the area-under-the curve (AUC), which was applied in a recently published four-weeks intervention study comparing vitD2 and vitD3 added to a malted drink at two daily levels, 5 µg or 10 µg vitD, which showed no difference in AUC between the groups receiving vitD2 and vitD3 [37].

The comparison of the effect of 25OH-D3 and vitD3 on the vitamin D status in human intervention studies designed with daily supplementation and same period for both compounds is limited to two studies. One study compared daily supplementation of 20 µg vitD3, 20 µg 25OH-D3, or 7 µg 25OH-D3 for 10 weeks [8]. Considering the change in vitamin D status from baseline until the end of the intervention, the conversion factor for 25OH-D3 to vitD3 was calculated to be 5. The AUC method was applied in the other study in postmenopausal women following daily supplementation for 15 weeks with 20 µg vitD3 or 25OH-D3 which resulted in 2–3 times higher area under the curve for the 25OH-D3 than that of vitD3 [9]. Our hypothesis was that the vitamin D metabolites would be able to maintain similar vitamin D status. We had to reject this hypothesis, and our estimated differences between the three vitamin D vitamers were not in line with previous results. No other studies have included three vitamin D active compounds.

Tripkovic et al. (2012) identified that the supplementation strategy should be taken into account in the effort to assess the relative differences between vitamers [6]. Therefore, it might be necessary to have two strategies in our efforts to assess the relative differences between the main vitamin D active compounds, vitD3, vitD2, and their 25-hydroxylated compounds. For clinical purposes, extreme bolus administration is necessary to eradicate a deficiency, but to estimate the contribution from the vitamin D metabolites in our food the conversion factor should be based on a more nutritional relevant daily intake.

Our aim was to estimate the efficacy of each of the vitamin active compounds present in our diet. Six weeks intervention period was used, based on the fact that the half-life of 25OH-D3 is estimated to be 15 days [17,38]. Furthermore, a stabilized vitamin D status after six weeks of daily supplementation have previously been observed following daily supplementation with 20 µg vitD3 in young and old men [36], 25 µg vitD3 in healthy adults [15], as well as for 5 µg, 10 µg, and 20 µg vitD3 in old women [39]. Owing to the limited time period for the whole study from October to March, only a four-week run-in period was used for the subjects to obtain their individual steady-state level at a daily supplementation of 10 µg vitD3. Furthermore, it was not possible to include a run-in period before each of the three intervention periods. However, the results showed no significant difference after the supplementation with vitD3 for six weeks following the intervention periods with vitD2 or 25OH-D3 or run-in with vitD3. The subjects were allowed to continue their usual diet, which secured a consistency in basal daily dietary intake of vitD for each of the subject in order not to interfere with the daily supplementation of 10 µg vitD. Nevertheless, the storage of vitD might influence the vitamin D status at the end of the intervention period. However, an investigation of labelled vitD3 supplementation in mini-pigs showed the contribution to vitamin D status from stored vitD3 declined from approximately 50 nmol/L to 5 nmol/L within a period of six weeks [40].

The reason we used a crossover design was to efficiently overcome the personal dependence on the individual differences, which also include the dependency on BMI [25] and genetic differences [41]. In the Caucasian Danish population, it has been shown that polymorphisms in *GC* and *CYP2R1* are associated with S-25OHD status [42]. Our hypothesis was that vitamin D status following each supplementation period would be the same for each of the individual subjects, and that the vitamin D status at end of each treatment would be independent of the start level. Each of the 12 subjects was randomly selected to receive supplementation in one of six different orders of vitD3, vitD2, and 25OH-D3. For the vitamin D status at the end of supplementation period with vitD3, we observed no dependency on start level of the given supplementation period, i.e., whether the subjects before this period had had six weeks of supplementation with vitD2 (lower vitamin D status) to six weeks of supplementation with 25OH-D3 (higher vitamin D status). This indicated that our assumption that each subject will have a vitamin D status at a certain level for a given supplementation level, which we name “steady state”, was true.

In addition to the effect on vitamin D status, daily supplementation of 20 µg 25OH-D3 compared to vitD3 in a four-month study in postmenopausal women showed that 25OH-D3, compared to vitD3 improved gait speed by 18%, but no effect could be demonstrated for trunk sway [43]. Furthermore, the 25OH-D3 treatment improved knee extension strength, decreased systolic blood pressure, and decreased more pronounced markers of innate immunity than did vitD3 [44].

Most intervention studies with vitamin D have focused on post-menopausal women or elderly women and men [45]. In studies aiming at investigating the increase in vitamin D status from supplementation, no differences were identified between young and old men [36], and no differences between gender has previously been described [8,28]. In this study we focused on the effect of dietary intake of vitamin D in a healthy population, and chose to focus on a homogeneous group of young men. The fact that we included only young males is a limitation in our study. The average vitamin D status following vitD3 supplementation was 55 nmol/L in the young males in the present study, whereas a previous study in healthy post-menopausal Danish women found the average vitamin D status to be 64 nmol/L [46]. Thus, we cannot rule out that gender and age differences in vitamin D status may occur. The number of subjects is a limitation in the assessment of the absolute difference between the vitamin D metabolites, while the strength of our study is the crossover design combined with the use of MS/MS-technique for quantification of vitamin D metabolites.

VitD3 is generally the primary form in food. However, vitD2 is the primary vitamin D form in wild mushrooms and vitD2 enriched button mushrooms approved for marketing in the EU [47], and 25OH-D3 is the main vitamin D metabolite in beef and liver [48]. Total content of vitamin D will be 30–50% lower with a factor 1.5 compared to the factor of 5 estimated from the content of vitD3 and 25OH-D3 [48].

Further studies are needed to verify our results that the potencies of vitD2 and 25OH-D3 are 0.44 and 1.5, respectively, compared to that of vitD3. We propose that the optimal study design is a crossover design including a run-in period, whereas the intervention period could be extended to eight or 12 weeks for each vitamin D metabolite to ensure a steady-state level.

## 5. Conclusions

In this study we hypothesized that vitamin D3, vitamin D2, and 25-hydroxyvitamin D3 affected vitamin D status equally. However, based on the obtained results, we rejected our hypothesis; the vitamin D status increased after supplementation with 25-hydroxyvitamin D3 and decreased after the supplementation with vitamin D2, compared to that after the supplementation with vitamin D3. Based on the estimation that 1 µg vitamin D3 per day provides an increase in vitamin D status of 1.96 nmol/L, the intake of vitamin D2 and 25-hydroxyvitamin D3 was converted to a similar content as vitamin D3 by multiplication by 0.44 and 1.5, respectively. To test if these conversion factors are correct, we propose a similar study to test the hypothesis that a daily supplementation with 10 µg vitamin D3, 23 µg vitamin D2, and 6.8 µg 25-hydroxyvitamin D3 will result in an equal vitamin D status. Our results contribute to the discussion on how to assess vitamin D activity based on chemical quantification of the individual vitamin D active compounds. Further investigations are needed to reach an international consensus on the contribution to vitamin D activity from the individual vitamin D metabolites.

## Figures and Tables

**Table 1:** Selected characteristics of the 12 male subjects, pre- and post-intervention.

Measure, Unit	Mean ± SD	Range
Age, year	23 ± 3	20–30
Height, cm	182 ± 6	174–194
Weight, kg		
Pre-intervention	76 ± 7	60–89
Post-intervention	77 ± 7	59–88
BMI, kg/cm^2^		
Pre-intervention	23 ± 2	19–27
Post-intervention	23 ± 3	19–28
Dietary vitamin D *, µg/day	1.1 ± 0.4	0.5–1.5
Dietary calcium *, mg/day	806 ± 361	431–1411

* Each subject filled-out the FFQ for vitamin D and calcium three times during the intervention. The values represent the mean and SD for the 12 subjects. The pooled SD within the subjects was 0.2 vitD/day and 285 mg calcium/day.

**Table 2:** Observed serum levels at baseline and after each treatment period (mean ± SD).

Compound in Serum	All Baseline	Treatment Group		
		VitD3	VitD2	25OH-D3
25OH-D3, nmol/L	54.6 ± 9.0	52.9 ± 8.5	32.3 ± 7.1	62.7 ± 11.5
25OH-D2, nmol/L	1.5 ± 1.0	2.2 ± 1.5	11.9 ± 3.1	2.1 ± 1.0
Total 25OH-D, nmol/L	56.1 ± 8.5	55.1 ± 8.9	44.2 ± 8.0	64.7 ± 11.2
VitD3, nmol/L	2.5 ± 1.5	2.0 ± 1.1	0.9 ± 0.8	0.8 ± 0.6
VitD2, nmol/L	0.04 ± 0.03	0.05 ± 0.04	0.3 ± 0.4	0.02 ± 0.01
PTH, pmol/L	3.2 ± 1.3	2.1 ± 0.7	2.8 ± 1.0	2.4 ± 0.9
Calcium, nmol/L	2.4 ± 0.1	2.5 ± 0.1	2.5 ± 0.1	2.5 ± 0.1

**Table 3:** Estimated level of vitamin D based on the model including the factors treatment and period, the covariate the baseline value and a random effect of person.

Level in Serum	Treatment for Six Weeks with 10 µg of			
	VitD3	VitD2	25OH-D3	*p* *
25OH-D3, nmol/L	52.2 (48.3; 56.3)	31.6 (29.3; 34.1)	61.6 (57.1; 66.5)	<0.001
25OH-D2, nmol/L	1.9 ^a^ (1.5; 2.3)	11.6 (9.2; 14.5)	1.9 ^a^ (1.5; 2.4)	<0.001
Total 25OH-D, nmol/L	54.4 (51.1; 58.0)	43.5 (40.9; 46.4)	63.8 (59.9; 67.9)	<0.001
VitD3, nmol/L	1.8 (1.3; 2.4)	0.7 ^a^ (0.5; 0.9)	0.6 ^a^ (0.5; 0.8)	<0.001
VitD2, nmol/L	0.04 (0.03; 0.05)	0.22 (0.15; 0.32)	0.02 (0.01; 0.03)	<0.001
PTH, pmol/L	2.0 ^a^ (1.7; 2.4)	2.6 ^b^ (2.2; 3.0)	2.2 ^ab^ (1.9; 2.6)	0.035
Calcium, nmol/L	2.5 ^a^ (2.4; 2.5)	2.5 ^a^ (2.4; 2.5)	2.5 ^a^ (2.4; 2.5)	0.958

* *p*-value is the overall treatment effect. Estimates sharing a letter in the same row are not significantly different at the 5% level.

## Supplementary Materials

The following are available online at http://www.mdpi.com/2072-6643/10/1/12/s1, Figure S1: Observed S-25OHD3 during the study for the 12 participants, Figure S2: Observed S-25OHD2 during the study for the 12 participants, Figure S3: Observed Total S-25OHD i.e., sum of S-25OHD2 and S-25OHD3 during the study for the 12 participants, Table S1: Vitamin D status before and after run-in period with daily supplementation of 10 μg vitD3. Sequence is a factor with 6 levels telling which treatment pattern the subject followed, Table S2: The uncertainty budget for the relative effectiveness for vitD2 compared to vitD3, Table S3: The uncertainty budget for the relative effectiveness for 25OH-D3 compared to vitD3.
