# Supplementary material for: Vitamin D Vitamers Affect Vitamin D Status Differently in Young Healthy Males
Source: Nutrients. 2017 Dec 23;10(1):12. doi: 10.3390/nu10010012 (PMC5793240; doi:10.3390/nu10010012)
Supplement: Supplementary file 1 [file nutrients-10-00012-s001.pdf]

nutrients

MDPI

Supplementary Materials for

Vitamin D vitamers affect vitamin D status differently in young healthy males

Jette Jakobsen[1*], Elisabeth Anne Wreford Andersen[2], Tue Christensen[1], Rikke Andersen[1] and Susanne Bügel[3]

[1] National Food Institute, Technical University of Denmark, 2800 Lyngby, Denmark
[2] Institute of Mathematics and Computer Science, Technical University of Denmark, 2800 Lyngby, Denmark
[3] Department of Nutrition, Exercise and Sport, University of Copenhagen, 1958 Frederiksberg, Denmark
* Author to whom correspondence should be addressed: jeja@food.dtu.dk (J.J);

3. Results

3.1. Characteristics of subjects

Table S1. Vitamin D status before and after run-in period with daily supplementation of 10 µg vitD3. Sequence is a factor with 6 levels telling which treatment pattern the subject followed.

Vitamin D status			Sequence		
Before run-in, nmol/L	After run-in, nmol/L	Difference, %			
62,1	57,9	-7	a	b	c
46,7	59,1	27	a	b	c
61,9	53,4	-14	a	c	b
51,8	63,7	23	a	c	b
48,4	46,2	-5	b	a	c
40,7	33,7	-17	b	a	c
59,4	52,4	-12	b	c	a
59,1	58,4	-1	b	c	a
80,8	64,8	-20	c	a	b
66,8	59,3	-11	c	a	b
69,1	61,0	-12	c	b	a
44,2	45,2	2	c	b	a

3.2. Effects of intervention with different vitamin D vitamers

In Figure S1, Figure S2 and Figure S3 we have plots of the observed vitamin D levels in the 12 subjects during the study. Period 0 is the baseline and Sequence is a factor with 6 levels telling which treatment pattern the subject followed. Each sequence was followed by 2 subjects (dotted and line).

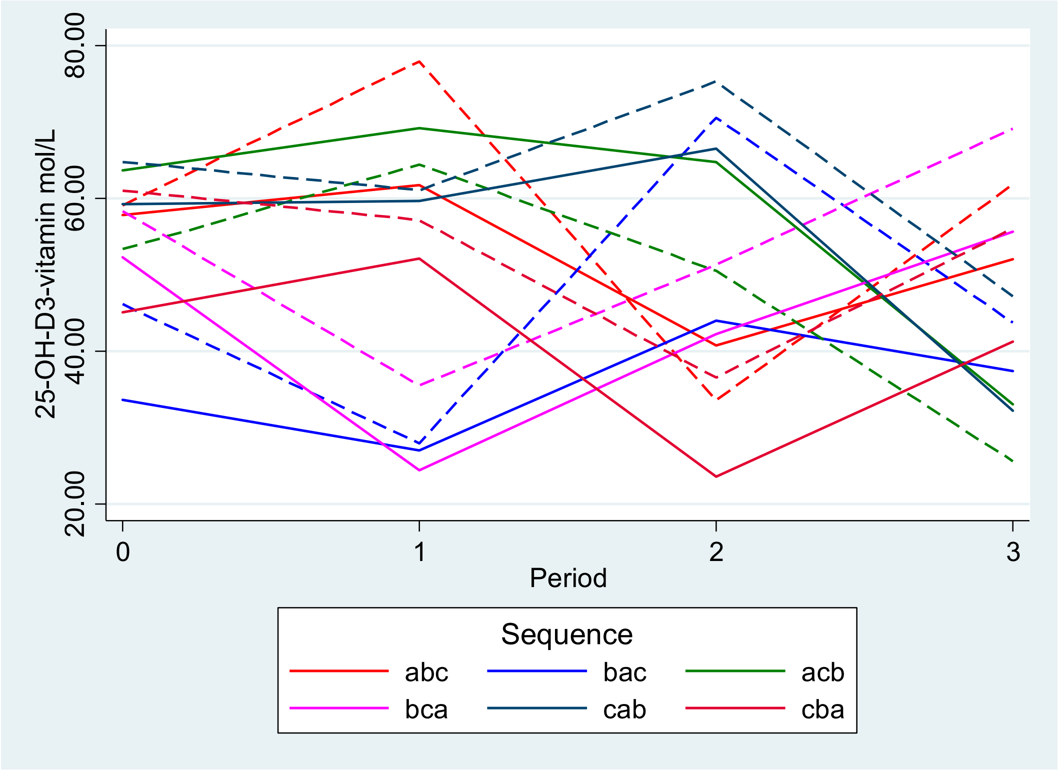

Figure S1: Observed S-25OHD3 during the study for the 12 participants.

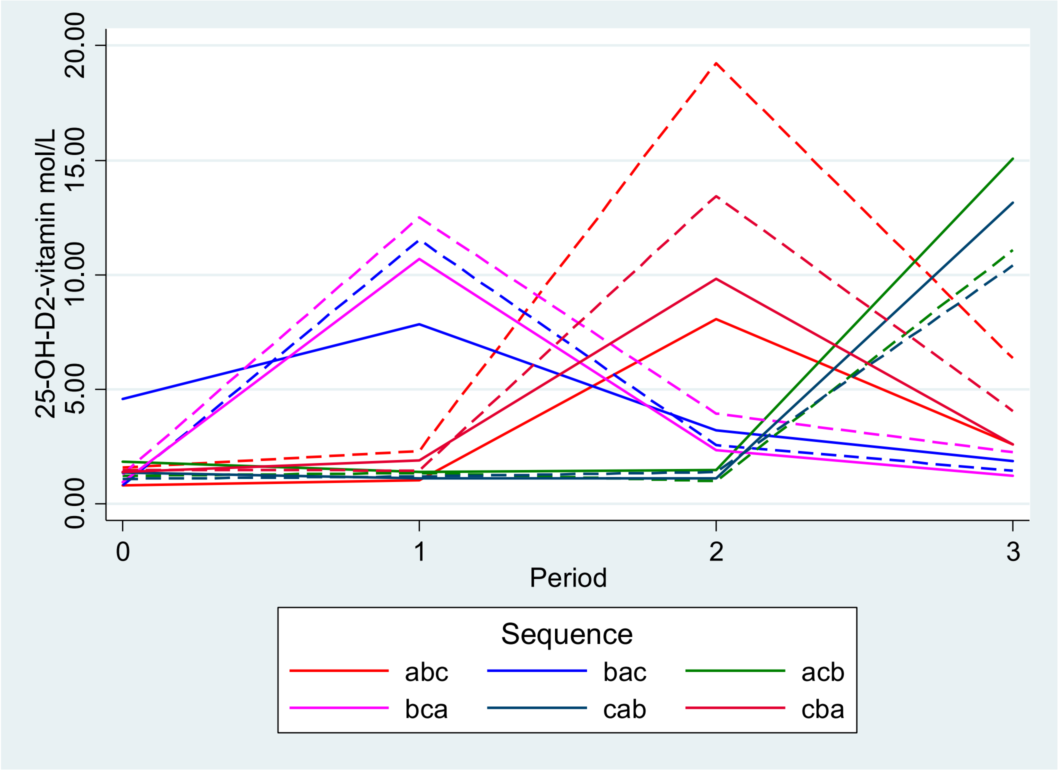

Figure S2: Observed S-25OHD2 during the study for the 12 participants.

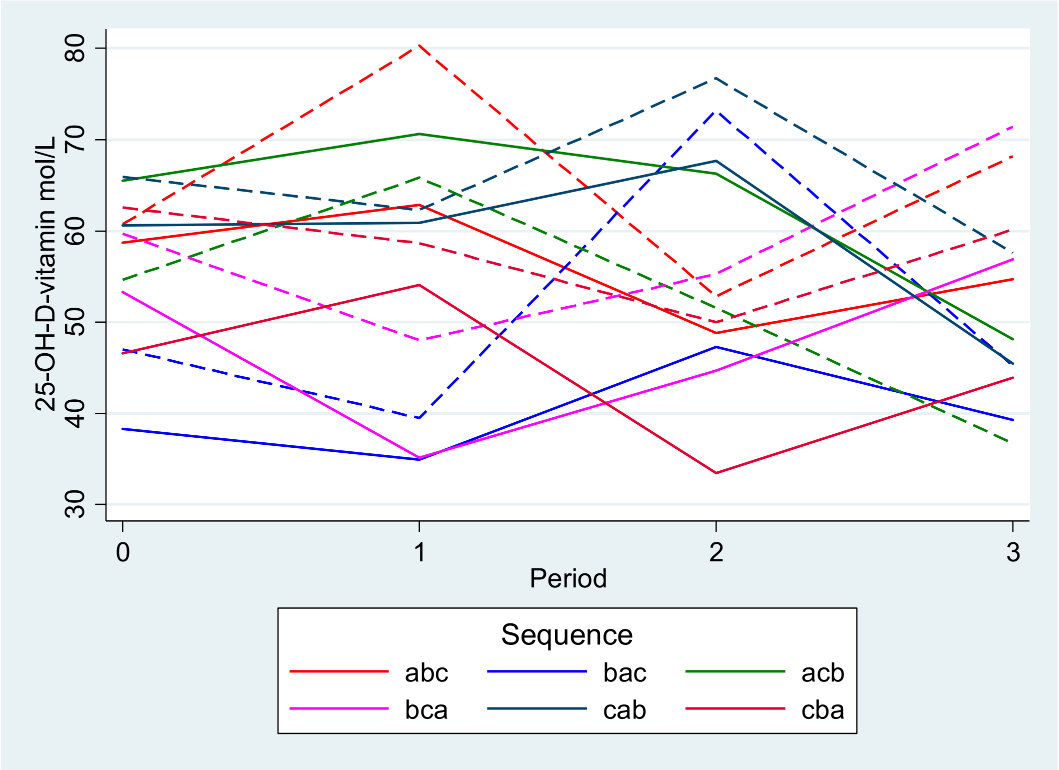

Figure S3: Observed Total S-25OHD i.e. sum of S-25OHD2 and S-25OHD3 during the study for the 12 participants.

3.3. Relative effectiveness of vitamin D vitamers to increase vitamin D status

The uncertainty budgets for the relative effectiveness for vitD2 compared to vitD3 and for 25OH-D3 compared to vitD3 are shown in Table S2 and Table S3, respectively. In the uncertainty budget the contribution from the uncertainty of content of vitamin D in the tablets is assumed to be included in the uncertainty of the measured vitamin D status.

Table S2 The uncertainty budget for the relative effectiveness for vitD2 compared to vitD3.

Parameter	Contributors	Level	Uncertainty	Comments
Effectiveness vitD2	Total vitD, end of vitD3 period	54.4 nmol/L	6.3%	Average of SD
	Total vitD, end of vitD2 period	43.5 nmol/L	6.3%	Average of SD
	Uncertainty for factor 1.96		9.0%	Estimated**
	Total uncertainty		***12.6%***	

Table S3 The uncertainty budget for the relative effectiveness for 25OH-D3 compared to vitD3.

Parameter	Contributors	Level	Uncertainty	Comments
Effectiveness 25OH-D3	Total vitD, end of vitD3 period	54.4 nmol/L	6.3%	Average of SD
	Total vitD, end of vitD2 period	63.8 nmol/L	6.3%	Average of SD
	Uncertainty for factor 1.96		9.0%	Estimated**
	Total uncertainty		***12.6%***	

** Factor 1.96 from Cashman et al. [27] is estimated to have similar uncertainty as the uncertainty on our primary data, as factor 1.96 is based on start and end level i.e. 9.0% which is the squareroot of the sum of the variance of the two contributors (Total vitD, end of vitD3 period and Total vitD, end of vitD2 period)
